# Increased Cell-Free DNA Plasma Concentration Following Liver Transplantation Is Linked to Portal Hepatitis and Inferior Survival

**DOI:** 10.3390/jcm9051543

**Published:** 2020-05-20

**Authors:** Felix Krenzien, Shadi Katou, Alba Papa, Bruno Sinn, Christian Benzing, Linda Feldbrügge, Can Kamali, Philipp Brunnbauer, Katrin Splith, Ralf Roland Lorenz, Paul Ritschl, Leke Wiering, Robert Öllinger, Wenzel Schöning, Johann Pratschke, Moritz Schmelzle

**Affiliations:** 1 Department of Surgery, Campus Charité Mitte and Campus Virchow-Klinikum, Charité—Universitätsmedizin, Corporate Member of Freie Universität Berlin, Humboldt-Universität zu Berlin, 13353 Berlin, Germany; felix.krenzien@charite.de (F.K.); alba.papa@charite.de (A.P.); christian.benzing@charite.de (C.B.); linda.feldbruegge@charite.de (L.F.); can.kamali@charite.de (C.K.); philipp.brunnbauer@charite.de (P.B.); katrin.splith@charite.de (K.S.); ralf-roland.lorenz@charite.de (R.R.L.); paul.ritschl@charite.de (P.R.); leke.wiering@charite.de (L.W.); robert.oellinger@charite.de (R.Ö.); wenzel.schoening@charite.de (W.S.); johann.pratschke@charite.de (J.P.); 2 Berlin Institute of Health (BIH), 10178 Berlin, Germany; bruno.sinn@charite.de; 3 Department of General, Visceral and Transplantation Surgery, Universitätsklinikum Münster, 48149 Münster, Germany; Shadi.Katou@ukmuenster.de; 4 Institute of Pathology, Charité—Universitätsmedizin, Corporate Member of Freie Universität Berlin, Humboldt-Universität zu Berlin, 10117 Berlin, Germany

**Keywords:** damage-associated molecular patterns, C-reactive proteins, systemic immune response, sepsis, portal hepatitis, liver transplantation, cfDNA, cell-free DNA

## Abstract

Donor organ quality is crucial for transplant survival and long-term survival of patients after liver transplantation. Besides bacterial and viral infections, endogenous damage-associated molecular patterns (DAMPs) can stimulate immune responses. Cell-free DNA (cfDNA) is one such DAMP that exhibits highly proinflammatory effects via DNA sensors. Herein, we measured cfDNA after liver transplantation and found elevated levels when organs from resuscitated donors were transplanted. High levels of cfDNA were associated with high C-reactive protein, leukocytosis as well as granulocytosis in the recipient. In addition to increased systemic immune responses, portal hepatitis was observed, which was associated with increased interface activity and a higher numbers of infiltrating neutrophils and eosinophils in the graft. In fact, the cfDNA was an independent significant factor in multivariate analysis and increased concentration of cfDNA was associated with inferior 1-year survival. Moreover, cfDNA levels were found to be decreased significantly during the postoperative course when patients underwent continuous veno-venous haemofiltration. In conclusion, patients receiving livers from resuscitated donors were characterised by high postoperative cfDNA levels. Those patients showed pronounced portal hepatitis and systemic inflammatory responses in the short term leading to a high mortality. Further studies are needed to evaluate the clinical relevance of cfDNA clearance by haemoadsorption and haemofiltration in vitro and in vivo.

## 1. Introduction

Liver transplantation continues to be the optimal treatment for various liver diseases. Transplant patients have an excellent long-term survival rate of ~50% over 20 years and often experience a good quality of life [1,2,3]. However, major clinical problems include the selection and allocation of donor organs in the face of an ageing society and organ scarcity [4,5]. To increase the donor pool, donation after cardiac death (DCD) has been introduced in some countries alongside standard donation after brain death (DBD) [6]. Another approach is to expand the donor criteria in order to decrease mortality on organ waiting lists. Extended criteria include prolonged ischemia, increased donor age, but also the use of older DCD donors [7]. The negative impact of marginal organs on survival is well known, although the molecular mechanisms remain uncertain.

Besides allogeneic stimulation of the organ recipient’s immune system, so-called damage-associated molecular patterns (DAMPs) can activate the innate and adaptive immune systems [8,9]. Cell-free DNA (cfDNA) can mediate a proinflammatory effect upon cell-death, either by necrosis, apoptosis, or active release of neutrophil extracellular traps. Endogenous DNA molecules activate inflammatory cascades in the absence of pathogen ectoreceptors as well as cytoplasmic receptors [10,11]. The most well-understood DNA sensors are Toll-like receptor 9 (TLR9), which are absent in melanoma 2 (AIM2) and cyclic GMP-AMP synthase (cGAS) [12]. 

Donor cfDNA has been proposed as a potential biomarker [13]. However, the effects of DBD characteristics on cfDNA release and the role of cfDNA in liver transplant recipients has hardly been investigated. Herein, we assessed the hypothesis that cfDNA is increased in transplant recipients following liver transplantation due to ischemic reperfusion injury (IRI). Furthermore, we demonstrated that high levels of cfDNA are associated with inflammation, portal hepatitis and inferior survival. In addition, we investigated the capacity of cfDNA clearance by haemoadsorption and haemofiltration in vitro and in vivo. Our findings provide novel insights of high clinical relevance, into the relationship between cfDNA, inflammation and survival following liver transplantation. Finally, haemofiltration is proposed as a possible anti-inflammatory therapy that might improve the outcome by removing the cfDNA.

## 2. Experimental Section

### 2.1. Approval

This retrospective analysis of prospectively collected blood samples, tissue samples and clinical data was conducted at the Department of Surgery, Campus Charité Mitte and Campus Virchow-Klinikum, Charité—Universitätsmedizin, Berlin, Germany, and was officially approved by the local ethics committee (EA2/148/12). 

### 2.2. Surgical Procedure and Immunosuppression

According to the guidelines of the German Medical Association, only DBD donors but not DCD donors are allowed in Germany [4]. Livers of DBD donors were allocated by Eurotransplant according to blood group compatibility and MELD score. Fifty patients who underwent orthotopic liver transplantation between January 2013 and May 2014 were included in the study. Surgery was performed as described previously by our group [14]. Patients younger than 18 years and combined liver-kidney transplantation were excluded from the study. A standard regime for immunosuppression was carried out using a calcineurin-inhibitor (tacrolimus more often than cyclosporin A) and low-dose steroids with or without supplementation of mycophenolate mofetil, especially in patients with reduced post-transplantation kidney function. Note, steroids were consequently tapered off and stopped completely at 3 months after liver transplantation.

### 2.3. Histology Examination

Liver biopsies were performed as part of clinical diagnostics, but per protocol biopsies were not performed. Only biopsies taken between postoperative day (POD) 6 and POD 14 were considered for analysis. A total of 21 liver biopsies were performed during this period. Patients with a rejection >1 according to the BANFF classification were excluded from the analysis (*n* = 6). Liver biopsies were evaluated based on haematoxylin and eosin (HE)-stained slides by a senior pathologist blinded to clinical or paraclinical information. The presence and quantity of neutrophils, eosinophils and lymphocytes was determined in the lobular (0 = none; 1 = single cells; 2 = loose infiltrate; 3 = dense infiltrate) and portal (0 = none; 1 = single cells; 2 = foci ≥3 cells) compartments. In addition, the presence of interface hepatitis was recorded (0 = none; 1 = mild; 2 = moderate; 3 = severe). Furthermore, bile duct inflammation was assessed (0 = no neutrophils; 1 = neutrophils within epithelium).

### 2.4. Blood Collection and Laboratory Analyses

Blood was drawn immediately after the operation, after the patient has arrived at the ICU, as well as on POD 1, 3 and 7. Laboratory measurements of clinical parameters were performed in a routine clinical laboratory. During automatic differentiation on a fully automated XN 1000 five-part haematology system (Sysmex, Wakinohama-kaigandori, Japan), neutrophil, immature granulocyte, eosinophil, basophil, lymphocyte, and monocyte cell classes were differentiated. Erythroblasts were also detected, and the leukocyte count was automatically detected. For cfDNA analysis, blood samples were collected in ethylenediaminetetraacetic acid (EDTA) tubes and centrifuged at 2000× *g* for 10 min within 1 h after collection. The plasma supernatant was then transferred to a fresh tube and frozen at ‒80 °C.

### 2.5. DNA Extraction

The DNA extraction method employed here has been described previously by our group [15]. Briefly, after thawing the samples at room temperature, plasma was centrifuged at 1000× *g* for 10 min to remove any remaining corpuscular/cellular components. A QIAamp Blood DNA Mini Kit (Qiagen, Veno, The Netherlands) was used for DNA isolation, which was performed according to the manufacturer’s protocol. After lysis of plasma samples with protease and ethanol, samples were centrifuged several times using a spin column. Subsequently, 50 µL elution buffer was added and samples were incubated for 10 min at room temperature. Samples were then centrifuged at 6000× *g* for 1 min to elute DNA from the column, and stored at ‒20 °C for at least 24 h.

### 2.6. Quantitative Real-Time PCR (qRT-PCR)

The detection of cfDNA was initially performed by qRT-PCR using a StepOnePlus real-time PCR system (Applied Biosystems, Massachusetts, WA, USA). Two DNA fragments, 90 and 222 bp long, respectively, were amplified (cfDNA 90 and cfDNA 222) [15]. Both fragments were amplified with the same forward primer (5′-TGCCGCAATAAACATACGTG-3′) and different reverse primers (cfDNA 90: 5′-GACCCAGCCATCCCATTAC-3′ and cfDNA 222: 5′-AACAACAGGTGCTGGGAGAGG-3′). Primer sets were selected to amplify two lengths of a multi-locus L1PA2 sequence as described in the literature [16]. L1PA2 is a human long interspersed element (LINE) that occurs throughout the human genome and is not gene-coding. Reactions contained 10 µL GoTaq qPCR Master Mix (Promega, Fitchburg, MA, USA), 8 µL nuclease-free water, 0.5 µL forward primer (10 µM) and 0.5 µL reverse primer (10 µM). DNA dissolved in 1 µL elution buffer was added, and the thermal cycling conditions were as recommended by the manufacturer. Negative controls were included on each PCR plate to exclude possible contamination. A standard curve was prepared to determine the absolute concentration of cfDNA. For this purpose, DNA was extracted from 200 µL whole blood as described above. Using the eluate, PCR was performed to obtain the corresponding fragment, and the concentration was measured with a Nanodrop 2000 microvolume spectrophotometer and fluorimeter (Thermo Fisher Scientific, Waltham, MA, USA). Next, we applied the Rapid PCR Cleanup Enzyme Set (New England Biolabs, Ipswich, MA, USA) to the amplified PCR product to degrade excess primers and dNTPs for purification of PCR products. The concentration was measured using the Nanodrop 2000 apparatus and a dilution series was placed in one row of the plate. The threshold cycle (Ct) value was determined for each dilution, and at least three dilutions (1:16,000, 1:32,000, 1:64,000, 1:128,000, 1:256,000 or 1:512,000) were measured for each reaction plate. The Ct value for each dilution was used to establish a standard curve for determining the cfDNA yield with each probe. The measurement was performed in duplicates.

### 2.7. Haemodialysis In Vivo

Patients were dialysed only in the context of acute kidney failure following liver transplantation. This decision was made as part of the clinical routine and was not an intervention of the present observational study. Only patients who received continuous veno-venous haemofiltration (CVVH) for at least 3 consecutive days within the first 7 postoperative days were grouped as “haemodialysis”.

### 2.8. Haemodialysis In Vitro

The in vitro experiment was carried out using a fully equipped haemodialysis instrument (Multifiltrate, Fresenius Medical Care, Bad Homburg, Germany). The venous leg of the dialysis was connected to 1000 mL 0.9% NaCl solution containing cfDNA that had been isolated as described above. The dialysate flow rate was 1000 mL/h and the blood flow rate was 50 mL/min. According to the setup, after 5 min, a sample was taken out of the tubing system (through which blood normally flows) before the filter (control), after the haemoadsorption Cytosorb column (CytoSorbents, New Jersey, NJ, USA), after the AV 1000s Ultraflux haemofilter (Fresenius Medical Care, Bad Homburg, Germany), and after both combined.

### 2.9. Statistical Analysis

Continuous variables are presented as the median and range, while categorical variables are presented as a percentage. For univariate analysis of categorical variables, the χ^2^ test (for trends, if applicable) or the exact Fisher’s test were employed. Continuous variables were assumed not to be normally distributed and were tested with the Kruskal–Wallis test or the Mann–Whitney *U* test for pairwise analyses. The Youden index was used to obtain a cut-off value for cfDNA 90 bp and 222 bp. This cut-off value has the highest specificity and sensitivity for distinguishing between a survivor and a non-survivor. The conditional forward Cox regression model was created with all significant influencing factors of the univariate analysis. Results of the Cox regression are reported as the hazard ratio (HR) and 95% confidence interval (95% CI). Factors were subsequently included in the multivariate cox regression model if the *p*-value was below 0.10 in univariate analysis. Overall, an alpha value of *p* < 0.05 was considered to show statistical significance.

## 3. Results

### 3.1. Clinical Course and cfDNA

Table 1 shows the baseline characteristics of the cohort (*n* = 50), and compares these characteristics between patients with low levels of cfDNA versus patients with high levels of cfDNA. Patient characteristics were equally distributed between both groups. To enable a categorical classification and to distinguish between cfDNA^high^ and cfDNA^low^, a cut-off was determined using the Youden index. For cfDNA 90 bp this cut-off was 68.4 ng/mL, and for cfDNA 222 bp it was 24.60 ng/mL. 

**Figure 1:**
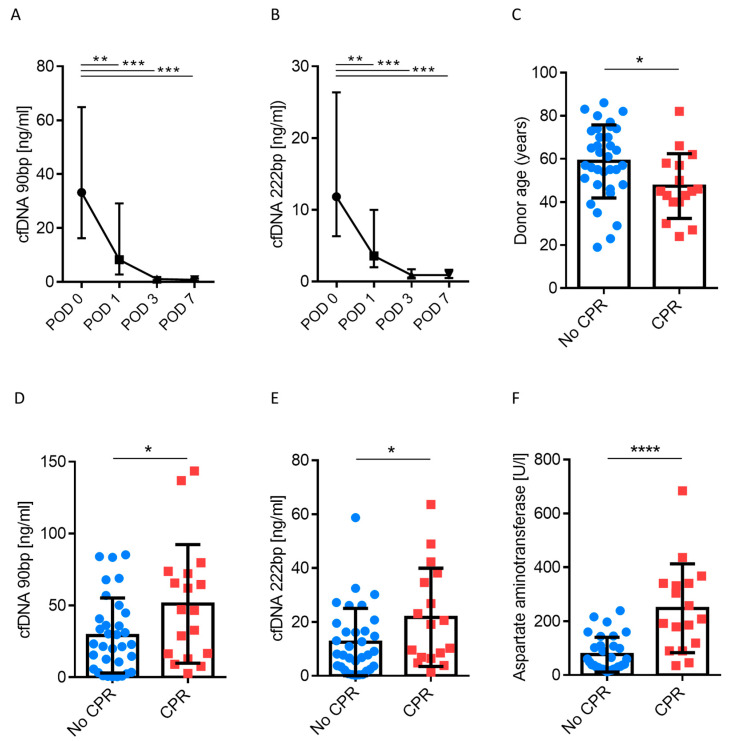
(**A**,**B**) Cell-free DNA (cfDNA) time course (90 bp and 222 bp L1PA2 element, human long interspersed element (LINE) sequence, non-coding) in serum in patients after liver transplantation (*n* = 50). Samples were taken when patients were admitted postoperatively to the intensive care unit. (**C**–**F**). Donor age and laboratory values analysed according to cardiopulmonary resuscitation (CPR). Mann–Whitney *U* test; * *p* < 0.05; ** *p* < 0.01; *** *p* < 0.001.

Next, we analysed the clinical course of patients with regard to plasma levels of cfDNA. As shown in Figure 1A,B, there was a significant decrease in cfDNA, by a factor of 47 (90 bp) and 13 (222 bp) within 7 days after liver transplantation (*p* < 0.05). cfDNA is typically released upon cell death, as is the case in IRI. Whole body IRI occurs in the course of cardiopulmonary resuscitation (CPR), and CPR is performed on a substantial subgroup of organ donors [17,18]. We therefore grouped transplant patients according to prior CPR of their organ donors and noted that recipient cfDNA 90 bp and 222 bp levels were twice as high when organs were sourced from donors who had previously undergone CPR (29.1 vs. 51.1 for 90 bp ng/mL and 12.5 vs. 21.7 for 222bp ng/mL, respectively, *p* < 0.05; Figure 1D,E, Supplementary Figure S1). Donors who underwent CPR were younger on average (Figure 1C); however, characterised by significantly elevated transaminases, when compared to donors who had not been resuscitated (Figure 1F). Notably, cfDNA levels were measured when patients arrived at the ICU after transplantation. Other donor characteristics such as laboratory parameters, length of stay on the ICU, or cause of death were not associated with a change cfDNA levels (Supplementary Figure S2, Supplementary Table S1). In addition, we investigated whether intra- and postoperative parameters or ischemia time were linked to cfDNA levels (Supplementary Table S2). While transfusion of blood products or ischemia time (warm and cold) had no influence on cfDNA, cfDNA was found to be correlated with elevated transaminases in the recipient after liver transplantation (*p* ≤ 0.001). 

In summary, cfDNA levels were elevated immediately after liver transplantation, but rapidly decreased over time within the first week after liver transplantation. Donors who had experienced CPR before DBD were younger, but exhibited increased transaminase level. Patients receiving these organs displayed elevated cfDNA levels and increased transaminase levels after transplantation, indicating cellular damage. 

### 3.2. Association between cfDNA and Survival 

**Figure 2:**
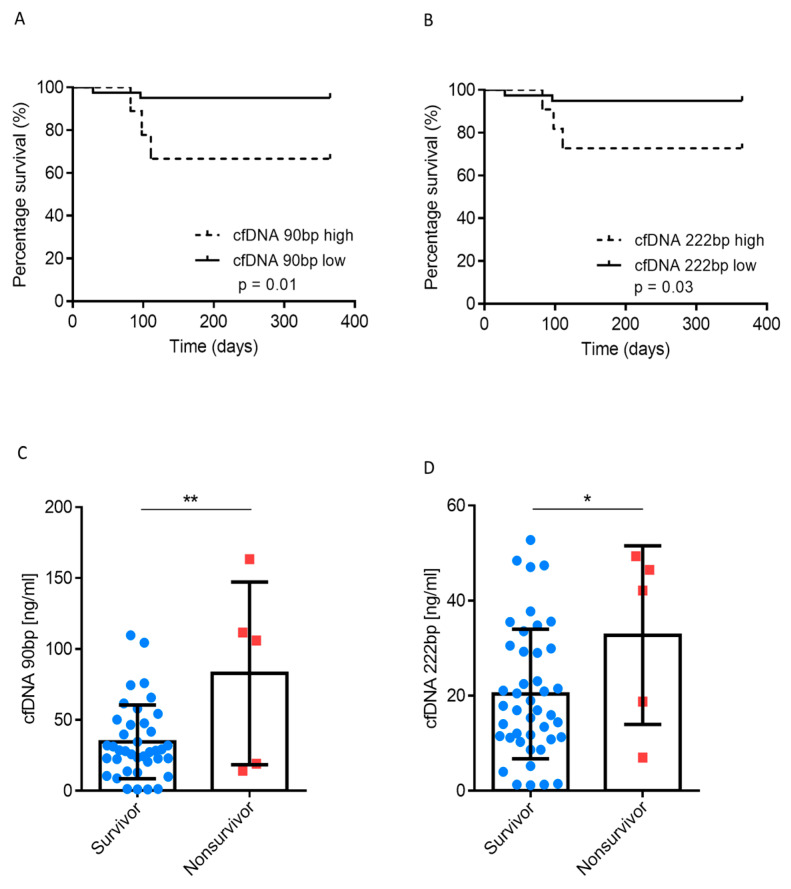
(**A**,**B**) cfDNA analysed according to survivors and non-survivors (1-year follow-up) (**C**,**D**). Patients analysed according to 1-year survival in high and low cfDNA groups. Mann–Whitney *U* test; Log-rank test (for survival); * *p* < 0.05; ** *p* < 0.01.

Next, we explored whether 1-year mortality was correlated with cfDNA. In total, there were five deaths within the investigated cohort, and none of these were related to any surgical vascular complications (e.g., hepatic artery thrombosis). Kaplan-Meier curves for 90 bp and 222 bp are shown in Figure 2A,B, respectively. Patients with cfDNA^low^ displayed a superior 1-year survival rate; the 1-year survival rate with cfDNA^low^ (90 bp) was 95%, compared with 66% for cfDNA^high^ (*p* = 0.01). Deaths with cfDNA^high^ occurred approximately 100 days after liver transplantation. All three patients with cfDNA^high^ who died after transplantation, died of sepsis due to liver abscess, while the two patients with lower cfDNA levels died of sepsis due to bile leakage. CPR of the donor alone did not exert any effect on 1-year survival (*p* = 0.735; Supplementary Figure S3). In fact, patients who died within 1 year had significantly higher cfDNA levels (Figure 2C,D). Univariate Cox regression analysis revealed that ischemic time (cold and warm), cfDNA, extrahepatic organ dysfunction (EROD) and labMELD score were of prognostic significance (Table 2). In multivariate analysis, only cfDNA was an independent prognostic factor for 1-year survival with a hazard ratio of 11.96 (95% CI, 1.11‒128.96).

In summary, cfDNA^high^ was associated with inferior 1-year survival rates and liver abscess followed by lethal sepsis. Moreover, cfDNA was an independent predictor for 1-year survival on multivariate analysis.

### 3.3. Inflammation in the Presence of High and Low cfDNA Levels

**Figure 3:**
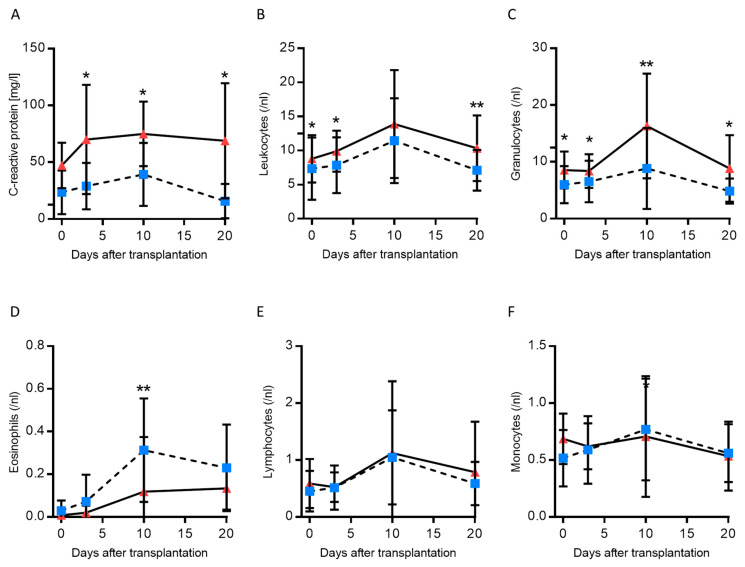
C-reactive protein, and differential blood count after liver transplantation (*n* = 50). (**A**–**F**). Patients were classified according to cfDNA concentration (L1PA2 element, 90 bp, LINE sequence, non-coding). Mann–Whitney *U* test; * *p* < 0.05; ** *p* < 0.01.

DNA fragments released upon apoptosis and necrosis are believed to mediate TLR-dependent (2-, 3- and 9-TLR), AIM2-dependent, and cGAS-dependent pro-inflammatory effects, as observed in IRI [10,12]. Therefore, we explored whether systemic inflammation and the number of immune cells in patients was correlated with high or low cfDNA levels (Figure 3). Note, the following analyses were performed using cfDNA 90 bp grouping measured on POD 0, since this measurement showed the highest correlation with survival. Tacrolimus trough levels were equally distributed in both groups (Supplementary Figure S4).

**Figure 4:**
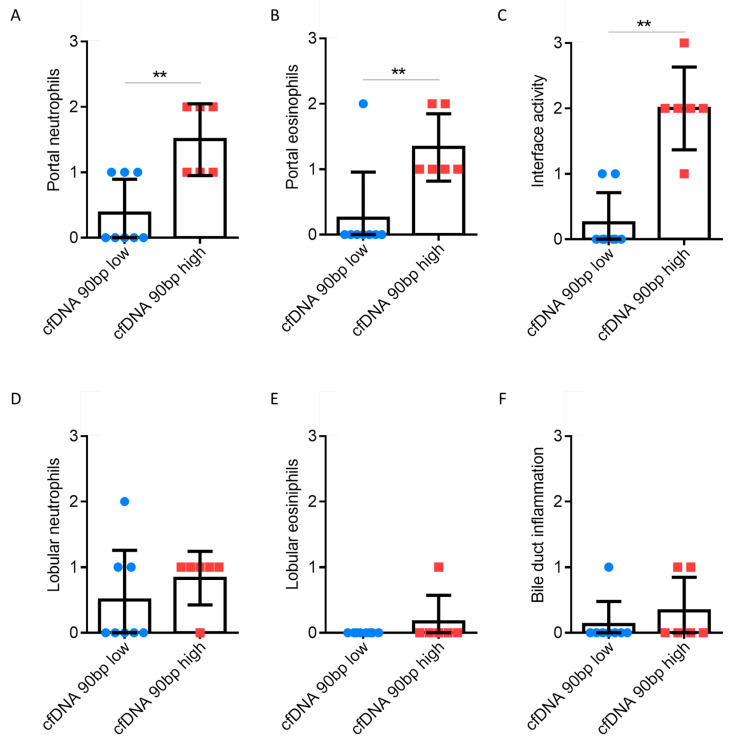
Evaluation of liver biopsies as part of clinical diagnostics based on haematoxylin and eosin staining. The presence and amount of neutrophils (**A**,**D**), eosinophils (**B**,**E**) was determined for patient groups with high and low cfDNA portal and lobular (L1PA2 element, 90 bp, LINE sequence, non-coding). Furthermore, interface activity (**C**) and bile duct inflammation (**F**) were determined. Samples with a rejection >BANFF 1 were excluded from the analysis. Mann-Whitney U test; ** *p* < 0.01.

**Figure 5:**
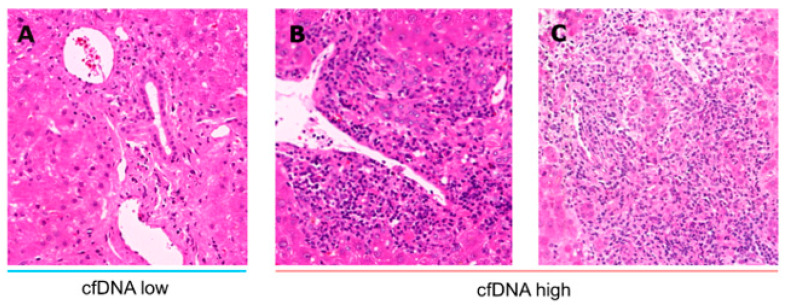
Representative liver biopsies for patients with high (**A**) and low (**B**,**C**) cfDNA levels. Liver biopsies were evaluated based on haematoxylin and eosin staining. Patients with high levels of cfDNA displayed florid portal inflammation and interface hepatitis. Samples with acute cellular rejection >BANFF 1 were excluded from the analysis.

C-reactive protein (CRP) and leukocytes were significantly increased over time in patients with cfDNA^high^. Specifically, granulocytes are the most abundant type of white blood cells, and these were significantly elevated at the measured time points, with no differences being evident for lymphocytes, monocytes or platelets (Figure 3 and Supplementary Figure S4). Although eosinophils in the blood of patients with cfDNA^high^ tended to be more abundant, statistical significance was only reached for one time point (POD 10, Figure 4D). Next, we explored liver transplant characteristics and local inflammatory reactions. Liver biopsies were analysed and are displayed in Figure 4. Patients with cfDNA^high^ levels displayed a florid portal inflammation and interface activity in biopsies. Lobular, but not portal lymphocyte infiltration was present (Supplementary Figure S5). Notably, patients with acute organ rejection were excluded from the analysis, while the distribution of acute rejection was similar for both groups (Supplementary Table S2). Representative histological slices are shown in Figure 5 for high and low cfDNA 90 bp groups. 

In summary, elevated CRP levels, leukocytosis and granulocytosis were observed in patients with cfDNA^high^ levels. In addition, these patients showed portal hepatitis and interface activity.

### 3.4. Clearance of Plasma cfDNA by Haemofiltration

**Figure 6:**
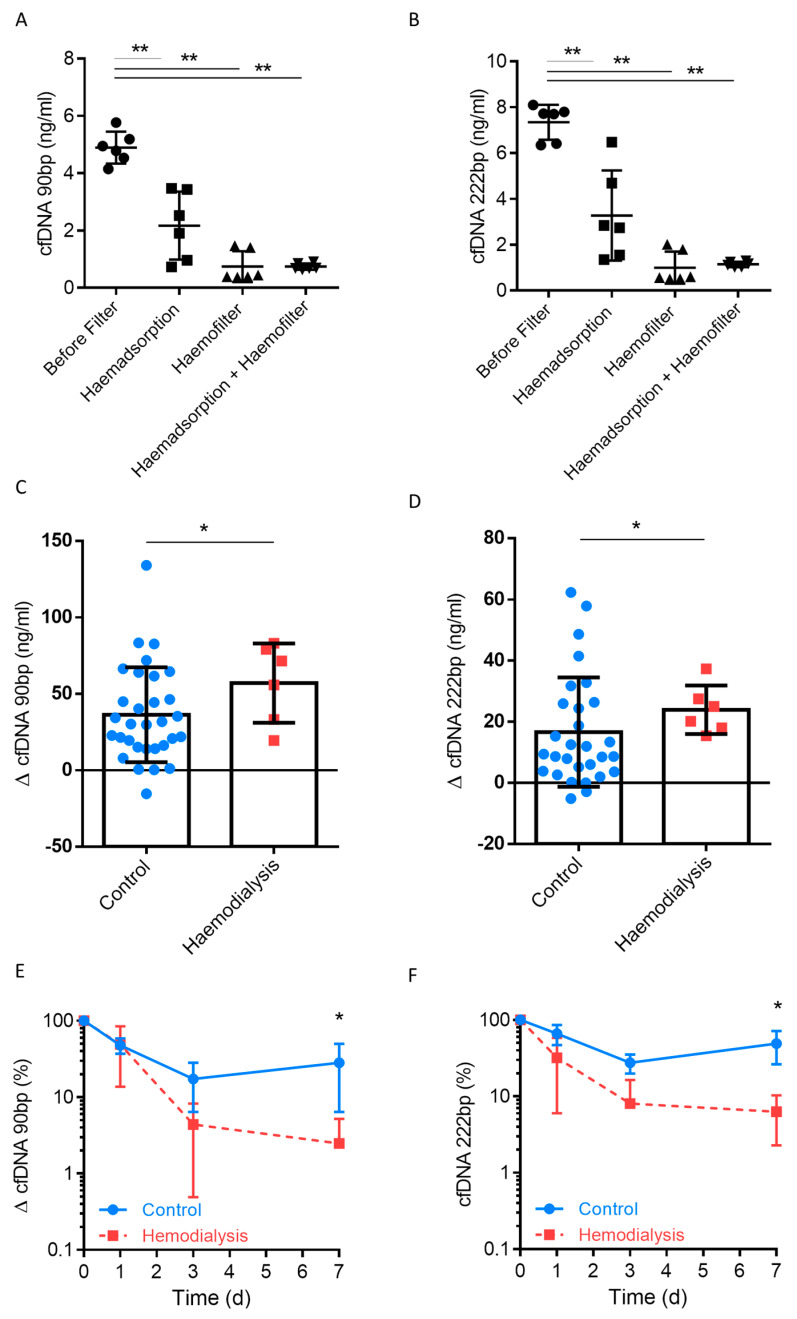
(**A**,**B**) A fully-equipped haemodialysis instrument was setup without connecting it to a patient, cfDNA was added to the arterial inflow, and cfDNA levels were determined in the fluid from the tubing system (where blood normally flows through) before the filter (control), after the Cytosorb haemadsorption column (CytoSorbents, New Jersey, NJ, USA), after the AV 1000s Ultraflux haemofilter (Fresenius Medical Care, Bad Homburg, Germany), and after both combined. Patients after liver transplantation (*n* = 50) grouped based on continuous veno-venous haemofiltration, which was performed on at least 3 days within the first 7 days postoperatively. Differences in cfDNA were subsequently determined. (**C**,**D**) Relative levels of cfDNA following haemodialysis. (**E**,**F**) Measurement of cfDNA in serum in 50 patients after liver transplantation (L1PA2 element, 90 bp and 222 bp, LINE sequence, non-coding). Mann–Whitney *U* test; * *p* < 0.05; ** *p* < 0.01.

DAMPs can be cleared by autophagy mediated by microtubule-associated protein 1 light chain 3 (LC3) and HMGB1 action, or by phagocytosis through DNA sensors [19]. In addition to the physiologically endogenous clearance of cell fragments such as cfDNA, they can be cleared by filtering or adsorbed by haemodialysis. To investigate this concept, we first set up a fully-equipped haemodialysis instrument without connecting it to patients, and added cfDNA to the arterial inflow. The results revealed a ~50% clearance through the haemoadsorption filter, while clearance rates spiked to >90% when a haemofilter was employed (Figure 6A,B). Thus, cfDNA levels were significantly decreased after only a single passage through haemoadsorption or haemofiltration. Next, we explored whether the use of routine CVVH resulted in clearance of cfDNA in the present liver transplantation cohort. Patients were classified according to the use of CVVH. In total, 8 patients underwent CVVH, which was performed on at least 3 days within the first 7 days postoperatively, while 4 patients underwent intermittent haemofiltration for less than 3 days. The results revealed a significant reduction in cfDNA 90 bp and 222 bp following CVVH when absolute values were analysed (Figure 6C,D). The trend for relative reduction can be seen in Figure 6E,F. The use of CVVH resulted in a more rapid decline in cfDNA during the postoperative course. The preoperative and postoperative creatinine level were not linked to cfDNA levels (Table 1, Supplementary Tables S1 and S2). In total, 4 patients needed renal replacement therapy before LT, and cfDNA did not show statistical difference between both groups (Supplementary Table S3). 

In summary, the use of a haemofilter or haemoadsorption can lead to cfDNA blood clearance when CVVH is applied. Using this technique, the initial high levels of cfDNA were significantly reduced following liver transplantation.

## 4. Discussion

**Figure 7:**
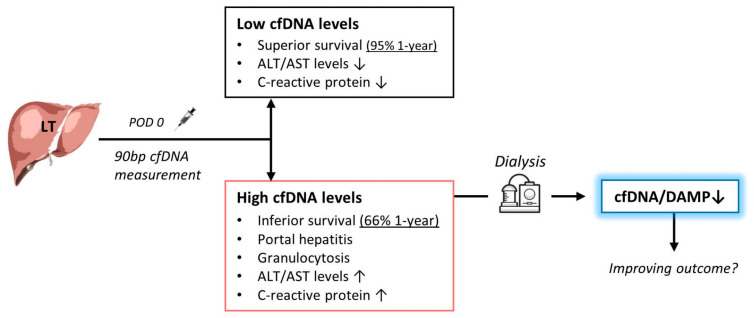
Schematic representation of the concept of using cell-free DNA (cfDNA) for the prediction of survival after liver transplantation and as a possible target in therapy. High levels of cfDNA in organ recipients are associated with a significantly poorer chance of survival. The cfDNA is a damage-associated molecular pattern (DAMP) that causes increased inflammation and lobular hepatitis in organ donors. Immediate measurement after liver transplantation can identify patients with high cfDNA levels. By using haemodialysis with haemofiltration and/or haemoadsorption, these DAMP could be filtered out of the blood to potentially improve the prognosis after liver transplantation.

In this study, we explored the possible implications of CPR prior to DBD and the relevance of cfDNA as a representative DAMP in the blood of liver transplant recipients. The results suggest that resuscitation is associated with increased cfDNA and transaminase levels due to liver-specific cell damage in the transplant recipients. Moreover, cfDNA^high^ patients after liver transplantation were associated with portal hepatitis, in addition to systemic inflammation with elevated granulocytes and CRP levels. Indeed, cfDNA^high^ patients showed inferior survival rates due to liver abscess and sepsis (Figure 7). More importantly, cfDNA was a strong independent predictor for 1-year survival on multivariate analysis. High cfDNA levels were noted early after transplantation, with the occurrence of abscesses several weeks later. Interestingly, the endogenous clearance of cfDNA was significantly reduced by applying CVVH in vitro and in patients. Whether this is indeed of therapeutic relevance appears to be worth addressing in future clinical trials. 

CPR before organ donation has already been investigated in a large registry trial by the United Network for Organ Sharing that analysed more than 54,000 liver transplantations after DBD donation [20], and CPR had no influence on transplant survival. Indeed, CPR can be performed prior to organ donation in brain-dead donors, in both an ambulatory setting or in hospital. This finding was reflected in our results, and in a previous solid organ transplantation study [17,18]. One explanation that cfDNA, but not CPR was linked to inferior survival would be that not every donor suffers from the same IRI after resuscitation. For example, the time of return of spontaneous circulation may vary greatly from patient to patient. Unfortunately, this was not sufficiently documented on the donor sheets of our study cohort and therefore an analysis was not possible. Indeed, CPR is known to cause warm ischemia, ischemia reperfusion damage and cell damage [21]. DAMPs are released upon cell damage, and this can lead to immune dysregulation [10]. Herein, we demonstrated that CPR of the donor led to a significant increase in cfDNA in the organ recipient. This is of importance since elevated cfDNA levels were linked to an increase in systemic inflammation responses in patients. Moreover, this group of patients showed a higher incidence of liver abscesses, which worsened the prognosis after liver transplantation.

In addition to the increase in cfDNA levels, there was also an increase in serum transaminases. AST and ALT are enzymes involved in amino acid metabolism that can be released by the liver, erythrocytes, heart, muscle and brain in response to cell damage. Transaminases differ from other so-called DAMPs such as DNA, RNA, HMGB1, S100 proteins, purines and saccharides, all of which are recognised by the adaptive or innate immune system, which triggers a non-infectious inflammatory response [8,9]. This does not occur with transaminases ALT and AST, although they are indicators of cell necrosis and apoptosis. However, there is no evidence that liver aminotransferases can be sensed by the immune system, or promote a proinflammatory state [22]. It is, therefore, reasonable to assume that the increased cfDNA levels are associated with liver cell damage indicated by the altered transaminases. Moreover, the transaminases were not a predictor of survival.

We found an increased number of granulocytes and eosinophils in liver biopsies of liver transplant patients with high levels of cfDNA. This is in line with increased numbers of neutrophils levels in the blood of those patients. In fact, sterile inflammation can occur undetected due to the release of DAMPs, leading to the recruitment of neutrophils [23]. Herein, we observed intrahepatic abscesses weeks after transplantation and time points of elevated cfDNA levels. The recruitment of neutrophils due to graft cell damage following CPR was speculated to lead to the development of intrahepatic abscesses. Indeed, the liver abscesses profoundly triggered the cause of fatal sepsis. Notably, none of the deaths in the present analysis was associated with vascular complications (e.g., occlusion of the hepatic artery). While the length of hospital stay did not show differences, patients with cfDNA^high^ stayed significantly longer in ICU. This appears plausible, since the mortality rate was increased in this patient group, which leads to the need for prior intensive care. 

The immunostimulatory effect of cfDNA has been demonstrated before. In human studies, cfDNA activates TNF-α mRNA expression and IL-6 production [24,25]. Moreover, nuclear but not mitochondrial cfDNA has been linked to reactive oxygen species ROS [26], and systemic ROS production appears to be caused by circulating phagocytes. In a recently published study of 21 patients receiving liver transplantation, NETosis was detected perioperatively [27]. However, most of the cfDNA was caused by graft cell death and not by NETosis. The authors concluded that cfDNA together with nucleosomes could contribute to the complex haemostatic balancing dynamics during liver transplantation. We have investigated the role of cfDNA in a previous clinical study with more than 80 patients in relation to non-alcoholic fatty liver disease (NAFLD) as a risk factor for fibrosis but also for cirrhosis. The cfDNA plasma concentration correlated with established non-invasive markers of NAFLD activity and severity 90 bp cfDNA levels were significantly higher in NAFLD patients compared to healthy controls (*p* = 0.014). Whether these results are related to cirrhosis-associated immune dysfunction could not be clarified [28].

The artificial clearance of DAMPs, and thus the elimination of proinflammatory molecules, is conceptually assumed to influence transplantation outcomes. Herein, we showed in vitro that a single passage through a haemofilter can achieve a higher clearance of cfDNA than haemoadsorption. In addition, we demonstrated that the use of CVVH after liver transplantation can significantly decrease cfDNA DAMPs. The application of CVVH was due to kidney injury and not due to increased levels of cfDNA. Therefore, it seems plausible, that CVVH was not linked to superior survival benefit, since these patient suffered from kidney failure. On univariate Cox regression analysis ischemic time (cold and warm), cfDNA, EROD (CVVH included) and labMELD score were of prognostic significance, while only cfDNA was an independent prognostic factor on multivariate analysis. The clinical relevance of clearing proinflammatory DAMPs remains controversial. Various randomised studies on the improvement of sepsis and acute renal failure report no improvement in survival despite a significant reduction of cytokines [29,30]. In both studies, the control group also received renal replacement therapy (high-volume versus low-volume haemofiltration). Comparison is therefore limited, since the control group also received machine clearance of DAMPs. This is a general methodological drawback that has not yet been addressed [31]. To overcome this, there is a need for a prospective trial to evaluate CVVH in patients without kidney failure but with high and low levels of cfDNA. 

Although the present human study reveals new findings, there are some limitations. The sample size (50 patients) was relatively small, and the applied subgrouping does not allow generalisation of the results, hence further exploration of cfDNA in liver transplantation patients is warranted. Clearance of cfDNA was recorded as part of this observational study. However, further evidence could be obtained by using an interventional study design with two treatment approaches (with and without CVVH). This was beyond the scope of the current analysis, but might be indispensable for assessing the use of CVVH after liver transplantation in cases of elevated cfDNA, irrespective of renal function.

In our qRT-PCR experiments, the non-coding L1PA2 segment was amplified. L1 elements account for almost 17% of the human genome [32]. The distribution of the L1PA2 sequence across all chromosomes increases the significance of measuring the true cfDNA concentration [16]. Differences in the measurement of cfDNA or L1PA2 repeats can vary, and may reflect polymorphism or differences in DNA methylation [32,33]. Furthermore, the concentration of cfDNA can depend strongly on sampling conditions. Zinkova and colleagues were able to show that the concentration of cfDNA (measured using the single copy gene 36B4) was 18-fold higher when serum samples were collected into tubes with clot activator and polymer gel [24]. The authors concluded that activation of coagulation and serum formation leads to NETosis, which increases cfDNA levels in serum. In the present study, samples were placed in EDTA tubes, and this may have impacted the measurements. However, this is true for all samples, and should therefore not influence our results.

## 5. Conclusions

In summary, patients receiving livers from resuscitated donors were characterised by high postoperative cfDNA levels. Those patients showed pronounced portal hepatitis and systemic inflammatory responses in the short term leading to a high mortality due to liver abscess and sepsis. Interestingly, the use of CVVH with filtration could significantly reduce cfDNA levels following liver transplantation. Whether the latter is of clinical importance remains uncertain and might be worth investigating in a prospective clinical trial of CVVH in patients without kidney failure but with high and low levels of cfDNA.

## Figures and Tables

**Table 1:** Baseline characteristics of the study cohort before liver transplantation.

Clinical Parameters	cfDNA Low (90 bp) *n* = 41	cfDNA High (90 bp) *n* = 9	*p*-Value
Age (years)	57 (22−70)	61 (28−65)	0.946
Sex			0.454
*Female*	15	5	
*Male*	26	4	
BMI (kg/m^2^)	25.0 (17.3−40.0)	28.6 (19.4−37.9)	0.393
Primary disease			
*Hepatocellular carcinoma*	14	2	0.573
*Cholangiocellular carcinoma*	2	1	0.844
*Alcoholic cirrhosis*	7	0	0.213
*Primary sclerosing cholangitis*	4	2	0.217
*Viral cirrhosis*	4	1	0.257
*Cryptogenic cirrhosis*	3	1	0.608
*Other*	8	2	0.699
labMELD	15 (6−40)	19 (6−40)	0.713
*International normalised ratio*	1.37 (0.97−5.3)	1.35 (0.99−2.14)	0.945
*Bilirubin total (mg/dL)*	2.1 (0.3−30.3)	4.7 (0.5−20.2)	0.838
*Creatinine (mg/dL)*	0.80 (0.47−4.67)	0.82 (0.48−4.0)	0.925
Sodium (mmol/L)	138 (128−144)	138 (129−142)	0.300

Listed laboratory results were determined before transplantation. cfDNA was determined in blood plasma taken immediately after transplantation (when patients arrived at the ICU). Continuous values are presented as median values and categorical values as numbers. Continuous variables were assumed to be non-normally distributed, and were tested using the Kruskal–Wallis test or Mann–Whitney *U* test for pairwise analyses. The χ^2^ test or Fisher’s exact test was used to test univariate differences between categorical variables.

**Table 2:** Prognostic factors determining 1-year survival

Clinical Parameters	Univariate HR (95% CI)	*p*-Value	Multivariate HR (95% CI)	*p*-Value
BMI kg/m^2^	0.92 (0.76−1.11)	0.402		
ICU (>10 days)	0.29 (0.05−1.76)	0.180		
Acute rejection (BANF ≥ 1)	62.86 (0.47−83803)	0.259		
AST recipient POD 0	1.00 (1.00−1.00)	0.807		
ALT recipient POD 0	1.00 (0.99−1.01)	0.780		
AST donor	1.00 (0.99−1.01)	0.754		
ALT donor	0.99 (0.98−1.01)	0.428		
Bilirubin donor	0.57 (0.11−2.88)	0.500		
Creatinine donor	0.98 (0.96−1.01)	0.392		
Lipase donor	1.00 (0.99−1.01)	0.663		
Resuscitation of donor	1.37 (0.23−8.19)	0.731		
labMELD	1.07(1.00−1.16)	0.043	1.056 (0.92−1.21)	0.422
cfDNAhigh/low 90 bp	8.34(1.39−50.03)	0.020	11.96 (1.11−128.96)	0.041
EROD	11.78 (1.32−105.82)	0.027	6.134 (0.18−203.99)	0.310
WIT (>45 min)	5.45 (0.91−32.71)	0.064	3.184 (0.30−33.95)	0.337
CIT (>6 h)	7.63 (0.85−68.35)	0.069	14.66 (0.95−226.59)	0.055

Univariate and multivariate analysis of factors influencing 1-year survival in liver transplant patients. Model for end-stage liver disease (MELD-Score); cell-free DNA (cfDNA), warm ischemia time (WIT), cold ischemia time (CIT), extrahepatic organ dysfunction (EROD), aspartate aminotransferase (AST), alanine aminotransferase (ALT). EROD was defined as requirement of de novo renal replacement therapy, reintubation/ventilation >7 days or cardiovascular event.

## Supplementary Materials

The following are available online at https://www.mdpi.com/2077-0383/9/5/1543/s1, Figure S1: The patients were grouped in cardiopulmonary resuscitation, Figure S2: Cell-free DNA (cfDNA) was determined in plasma from the liver transplanted patients (*n* = 50) and analyzed according to cardiopulmonary resuscitation (CPR), Figure S3: Patients were analyzed according 1-year survival grouped in CPR and no CPR of the donor, Figure S4: Presentation of the course of monocytes, platelets after liver transplantation (*n* = 50), Figure S5: Liver biopsies as part of clinical diagnostics were evaluated based on hematoxylin-eosin stained-tissue sections, Table S1: Baseline characteristics of organ donors, Table S2: Surgical and clinical parameters of the study cohort, Table S3: Preoperative renal replacement therapy.
