# Supplementary material for: Increased Cell-Free DNA Plasma Concentration Following Liver Transplantation Is Linked to Portal Hepatitis and Inferior Survival
Source: J Clin Med. 2020 May 20;9(5):1543. doi: 10.3390/jcm9051543 (PMC7291032; doi:10.3390/jcm9051543)
Supplement: Supplementary file 1 [file jcm-09-01543-s001.pdf]

Suppl. Table 1. Baseline characteristics of organ donors

**Clinical parameters**	**cfDNA low** *(90 bp)* *n = 41*	**cfDNA high** *(90 bp)* *n = 9*	****p-value***
Age (years)	56 (19–86)	50 (39–66)	0.181
Sex			0.722
Female	17	3	
Male	24	6	
BMI (kg/m^2)	26.1 (19.5–34.9)	27.7 (20.6–34.2)	0.451
**Reanimation** (yes/no)	**11/30**	**7/2**	**<0.001**
ROSC (min)	0 (0–125)	7 (0–45)	0.107
Brain death due to			
Intracranial bleeding	18	5	0.525
Traumatic brain injury	8	1	0.658
Thrombembolic event	8	0	0.178
Hypoxia	7	3	0.177
Microscopic liver fat (%)	5 (0–90)	0 (0–60)	0.427
Stay at ICU (days before donation)	3.5 (0–16)	1.5 (0–11)	0.095
Diuresis during last 24 h (ml)	3600 (1131–9800)	4939 (1860–6400)	0.420
Bilirubin total, mg/dl	0.57 (0.17–4.1)	0.64 (0.3–2.8)	0.924
Creatinine, mg/dl	1.0 (0.20–2.25)	0.86 (0.44–2.11)	0.705
Aspartate transaminase, U/l	90 (16–684)	145 (12–436)	0.643
Alanine transaminase, U/l	52 (4–484)	80 (20–828)	0.571

Continuous values are presented as median values and categorical values as numbers. Continuous variables were assumed to be non-normally distributed, and were tested using the Kruskal-Wallis test or the Mann-Whitney U test for pairwise analyses. The $\chi 2$ test or Fisher's exact test were used to test univariate differences between categorical variables.

Suppl. Table 2. Surgical and clinical parameters of the study cohort.

Clinical parameters	cfDNA low *(90 bp)* *n = 41*	cfDNA high *(90 bp)* *n = 9*	******p-value*
Surgical time, min	344 (235–557)	320 (266–424)	0.414
Cold ischemia time, min	526 (280–850)	592 (360–770)	0.264
Warm ischemia time, min	40 (25–58)	40 (21–60)	0.829
Red blood cell transfusion (intraop.)	6 (0–20)	4 (0–27)	0.837
Fresh frozen plasma (intraop.)	17 (0–57)	18 (13–59)	0.257
International normalized ratio	1.52 (1.17–293)	1.79 (1.33–2.27)	0.124
Bilirubin, mg/dl	3.7 (1.1–17.0)	4.23 (0.73–11.8)	0.905
Creatinine, mg/dl	0.95 (0.57–4.49)	1.19 (0.58–1.8)	0.780
**Aspartate transaminase, U/L**	**710 (182–7000)**	**2013 (1075–7000)**	**<0.001**
**Alanine transaminase, U/L**	**376 (48–3301)**	**1391 (706–2576)**	**0.001**
Acute rejection 1 year (yes/no)	20/21	7/2	0.114
BANF > 1	12	2	0.669
Length of stay (days)	28 (13–122)	29 (17–158)	0.436
**ICU stay (days)**	**8 (3–87)**	**11 (5–98)**	**0.027**
EROD (yes/no)	11/30	4/5	0.296
Death due to			
Vascular complication	0	0	
Liver abscess/sepsis (yes/no)	**2/37**	**3/6**	**0.004**
Bile leakage (yes/no)	2/39	0/9	1.000

Listed laboratory results were determined simultaneously alongside cfDNA when patients arrived at the ICU after transplantation. Continuous values are presented as median values and categorical values as numbers. Continuous variables were assumed to be non-normally distributed, and were tested using the Kruskal-Wallis test or the Mann-Whitney U test for pairwise analyses. The $\chi 2$ test or Fisher's exact test were used to test univariate differences between categorical variables.

Suppl. Table 3: Preoperative renal replacement therapy

	**Preoperative RRT**<br>n = 4	**No RRT**<br>**n = 46**	******p-value*
cfDNA 90bp, ng/ml	20.62 (5.77−79.73)	30.2 (0.47−143.6)	0.769

cfDNA was determined when patients arrived at the ICU after transplantation. Continuous values are presented as median values and were assumed to be non-normally distributed, and were tested using the Kruskal-Wallis test or the Mann-Whitney U test for pairwise analyses. Renal replacement therapy (RRT)

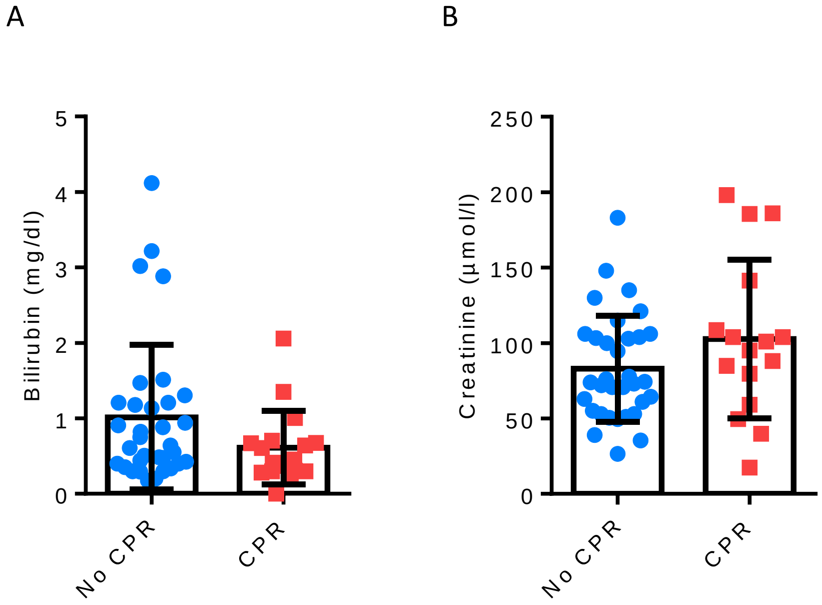

Suppl. Figure 1: The patients were grouped in cardiopulmonary resuscitation.Statistics: Mann-Whitney U-Test; Log-rank test (for survival); * P <0.05; ** P < 0.01; *** P < 0.001.

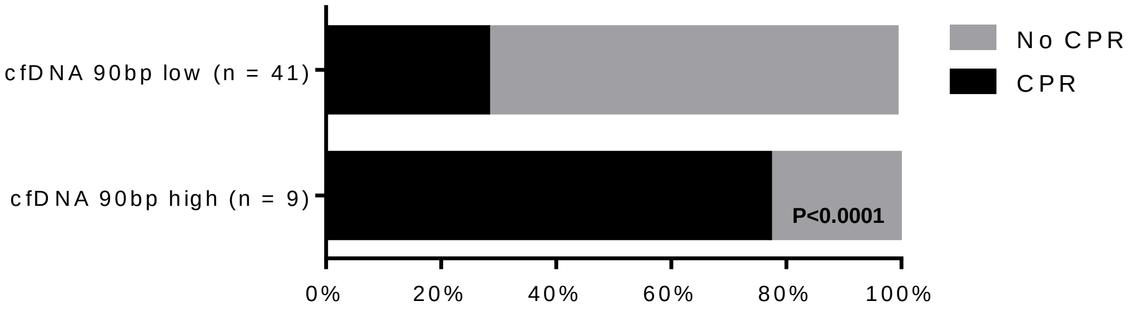

Suppl. Figure 2: Cell-free DNA (cfDNA) was determined in plasma from the liver transplanted patients (n = 50) and analyzed according to cardiopulmonary resuscitation (CPR). Samples were taken, when patients were admitted postoperatively to the intensive care unit. L1PA2 element non-coding sequence 90bp was determined by qPCR (L1PA2 element, 90bp, LINE sequence, non-coding). Statistics: Mann-Whitney U-Test; * P <0.05; ** P < 0.01; *** P < 0.001.

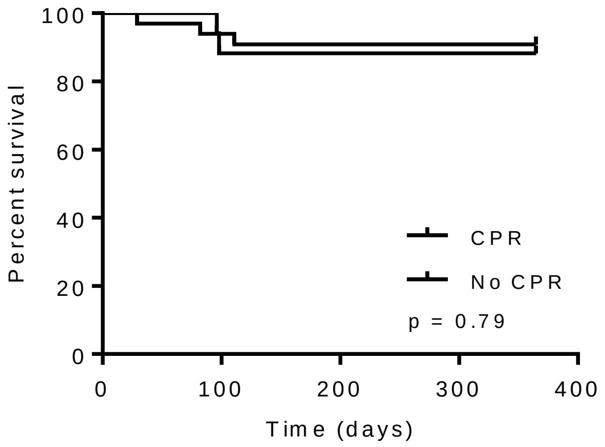

Suppl. Figure 3: Patients were analyzed according 1-year survival grouped in CPR and no CPR of the donor. Statistics: Mann-Whitney U-Test; Log-rank test (for survival); * P <0.05; ** P < 0.01; *** P < 0.001.

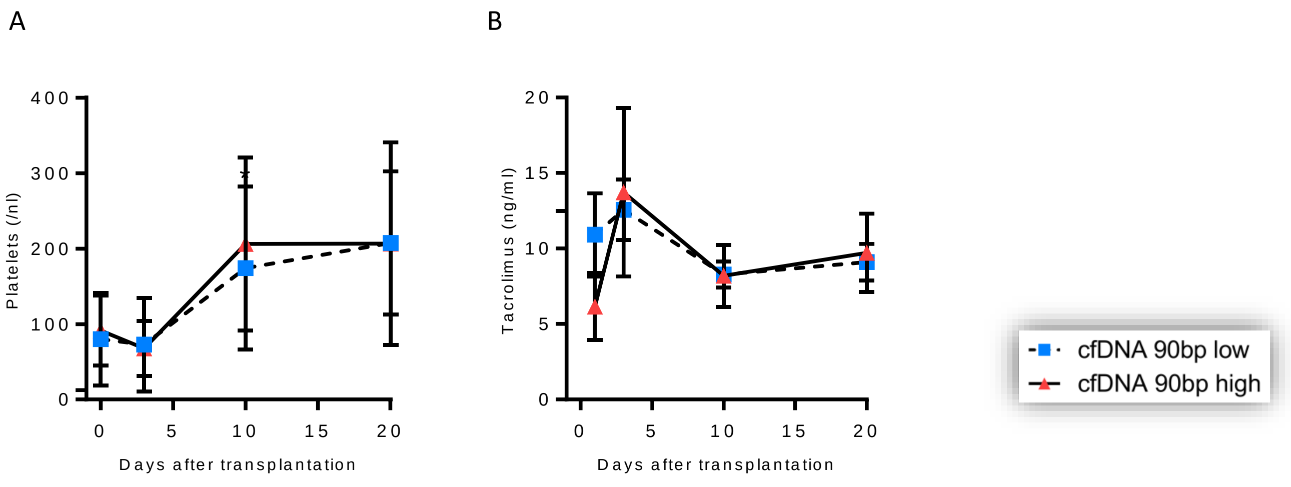

Suppl. Figure 4: Presentation of the course of monocytes, platelets after liver transplantation (n = 50). Patients were classified according to cfDNA concentration (L1PA2 element, 90bp, LINE sequence, non-coding). Statistics: Mann-Whitney U-Test; * P <0.05; ** P < 0.01; *** P < 0.001

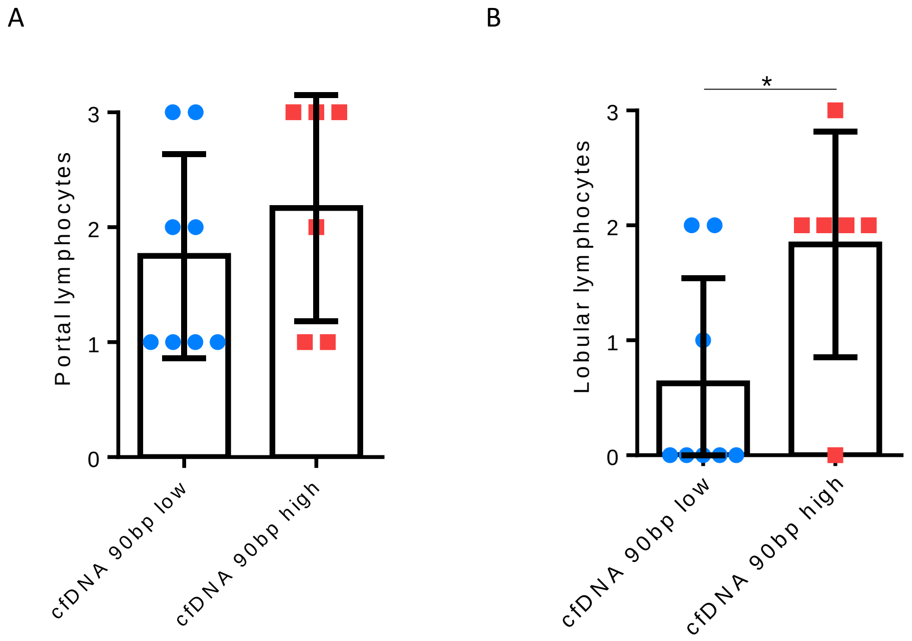

Suppl. Figure 5: Liver biopsies as part of clinical diagnostics were evaluated based on hematoxylin-eosin stained-tissue sections. The presence and quantity of lymphocytes was determined and patients were grouped in high and low cfDNA levels (L1PA2 element, 90bp, LINE sequence, non-coding). Note, rejection > BANFF 1 were excluded. Statistics: Mann-Whitney U-Test; * P <0.05; ** P < 0.01; *** P < 0.001
